# Individualized luteal phase support after fresh embryo transfer: unanswered questions, a review

**DOI:** 10.1186/s12978-021-01320-7

**Published:** 2022-01-22

**Authors:** Jing ZHAO, Jie HAO, Yanping LI

**Affiliations:** 1 Reproductive Medicine Center, Xiangya Hospital, Central South University, 87 Xiangya Road, Changsha City, Hunan Province People’s Republic of China; 2 Clinical Research Center For Women’s Reproductive Health In Hunan Province, Hunan, People’s Republic of China

**Keywords:** Individualized luteal phase support, Assisted reproductive technology, Progesterone

## Abstract

**Background:**

Luteal phase support (LPS) is an important part of assisted reproductive technology (ART), and adequate LPS is crucial for embryo implantation. At present, a great number of studies have put emphasis on an individualized approach to controlled ovarian stimulation (COS) and endometrium preparation of frozen- thawed embryo transfer (FET); However, not much attention has been devoted to the luteal phase and almost all ART cycles used similar LPS protocol bases on experience.

**Main body:**

This review aims to concisely summarize individualized LPS protocols in fresh embryo transfer cycles with hCG trigger or GnRH-a trigger. The PubMed and Google Scholar databases were searched using the keywords: (luteal phase support or LPS) AND (assisted reproductive technology or ART or in vitro fertilization or IVF). We performed comprehensive literature searches in the English language describing the luteal phase support after ART, since 1978 and ending in May 2019. Recent studies have shown that many modified LPS programs were used in ART cycle. In the cycle using hCG for final oocyte maturation, the progesterone with or without low dose of hCG may be adequate to maintain pregnancy. In the cycle using GnRH-a for trigger, individualized low dose of hCG administration with or without progesterone was suggested. The optimal timing to start the LPS would be between 24 and 72 h after oocyte retrieval and should last at least until the pregnancy test is positive. Addition of E_2_ and the routes of progesterone administration bring no beneficial effect on the outcomes after ART.

**Conclusions:**

Individualized LPS should be applied, according to the treatment protocol, the patients’ specific characteristics, and desires.

## Plain language summary

Luteal phase support (LPS) is an important part of assisted reproductive technology (ART). In the cycle using hCG for final oocyte maturation, the progesterone with or without low dose of hCG may be adequate to maintain pregnancy. In the cycle using GnRH-a for trigger, individualized low dose of hCG administration with or without progesterone was suggested. The optimal timing to start the LPS would be between 24 and 72 h after oocyte retrieval and should last at least until the pregnancy test is positive. Addition of E_2_ and the routes of progesterone administration bring no beneficial effect on the outcomes after ART. Individualized LPS should be applied, according to the treatment protocol, the patients’ specific characteristics, and desires.

## Background

Forty-three years have passed since the first tube baby was born in 1978. So far, several kinds of ovary stimulation protocols and endometrium preparation protocols have been put forward according to the individual’s self-conditions and personal desires, in order to gain high quality embryos and enhance the endometrial receptivity. At present, gonadotropin- releasing hormone agonists (GnRH-a) or antagonist (GnRH-ant) have been used in controlled ovary stimulation (COS) for preventing premature luteinizing hormone (LH) elevation [1]. The LH level was still suppressed 9 days after the GnRH-a was discontinued with long GnRH-a protocol [2, 3]. GnRH-ant protocol with GnRH-a trigger is especially prone to premature luteolysis, resulting in significantly decreased pregnancy rate [4, 5]. The luteal function was assumed to be impared in COS cycles with GnRH-a or GnRH-ant [6].

Luteal phase deficiency (LPD) has been due to reduced luteal support from pituitary LH, decreased steroid production in the corpus luteum (CL) and/ or premature luteolysis [7]. LPD is characterized by insufficient or inappropriate progesterone production. LPD is evident among women receiving the COS treatment using the GnRH analogue. This inevitably undermines the ability to successfully establish and maintain pregnancy. If there was no luteal phase support (LPS) after in vitro fertilization (IVF), the luteal phase length became shortened and bleeding often occurs early [8].

Hence, LPS is necessary for both luteal insufficiency and early pregnancy. A meta-analysis [9] and two Cochrane systematic reviews [10] confirmed that LPS improves the IVF pregnancy outcome. In clinical practice, whether it is a COS cycle or a frozen embryo transfer (FET) cycle, the administration of progesterone is routine for LPS. However, LPS does not have so many choices as the individualized COS protocols and endometrium preparation protocols.

Currently, an individualization of LPS has not been yet well implemented. The present review aim to summarize the characteristics of different LPS and the possibilities of individualized LPS.

## Methods

This review aimed to summarize individualized LPS protocols in fresh embryo transfer cycles with hCG trigger or GnRH-a trigger. For this, the PubMed and Google Scholar databases were searched using the keywords: (luteal phase support or LPS) AND (assisted reproductive technology or ART or in vitro fertilization or IVF). We performed comprehensive literature searches in the English language describing the luteal phase support after ART, since 1978 and ending in May 2019. After reading abstract and/or full texts and synthesizing relevant evidence, literature was oraganized thematically. Themes were discussed and decided opon by all three authors.

### Characteristic of luteal phase in natural cycle or ovary stimulated cycle

#### Characteristic of luteal phase in natural cycle

The corpus luteum (CL) produces different hormones, mainly including progesterone. Progesterone stimulates transformation of the endometrium, preparing for embryo implantation.

The LPD can result in infertility or miscarriage, because of insufficient progesterone during embryo implantation or early pregnancy. The LPD, as one of the possible factors for subfertility, was first described by Jones [11]. Endometrial biopsy is considered the ‘gold standard’ for the disgnosis of LPD. Published researches reported that the incidence of LPD in infertile women was 5–32.5% [12], and based on endometrial histology, the incidence of LPD in infertility women with natural ovulation was about 8.1% [13].

Although in most cases, endogenous progesterone may be sufficient for embryo implantation, there still some women who receiving natural cycle (NC)-IVF/FET may have endometrial abnormalities due to the presence of LPD, reducing implantation and pregnancy rates. There is a question: whether progesterone supplementation should be performed on all normal ovulation women received NC-IVF/FET treatment. So far, studies have shown that young women with normal ovarian reserve and normal BMI cannot gain benefitial from LPS with progesterone during the natural cycle.

#### Characteristic of luteal phase of ovary stimulated cycle

Ovarian stimulation (OS) aimed to increase the number of available oocytes. Although OS improved the efficacy of assisted reproductive technology (ART), it has changed the normal function of CL as well, involving multiple mechanisms.

The primary cause of LPD is the super-physiologic level of E2 produced in the OS, which reaches or even exceeds 10 times the level encountered in the natural cycle. Similarly, under the influence of trigger with hCG or GnRH-a, the level of progesterone produced by multiple CLs greatly exceed the normal level encountered in the natural cycle. The function of the hypothalamopituitary complex was interfered with elevated E2 and progesterone in the early LP, resulting in impaired LH secretion [14].

Secondly, the aim of GnRH-a in ART is to prevent premature elevation of LH and progesterone. The inhibition effect can lasting for 2–3 weeks after the end of GnRH-a treatment, resulting in decresed secretion of progesterone while the hCG stimulation effects disappear, which result in the “luteal gap” between the stimulation effects of exogenous hCG trigger and endogenous hCG after pregnancy [15, 16]. The production of endogenous progesterone might be reduced during this luteal gap, causing potential detrimental effect on early pregnancy.

In the GnRH-ant cycle, GnRH-a can be used for trigger in women who at a high risk of ovarian hyperstimulation syndrome (OHSS) [17]. After GnRH-a trigger, GnRH-a combined with the GnRH receptor, and caused the LH and FSH surge, leading to oocyte final maturation and ovulation [18]. But, this LH/FSH surge is shorter compared with the natural cycle. Under this condition, granulosa cells (GCs) cannot luteinized completly, leading to impaired secretion of LH and shortened lifespan of CL [19]. So, the luteal phase will be luteolysis and insufficient [20]. Previous studies have suggested that severe luteolysis would be developed within about 5 days after trigger with GnRH-a [21]; recently, it has been clearly showed that luteolysis is patient specific [22].

It has been shown in a basic study that GCs after COS, especially with the GnRH-a trigger, has lower survival rates in vitro and has lower levels of LH receptor as well as down-regulated expression of anti-apoptotic genes. Consequently, these GCs undergo apoptosis earlier than that of NC cycle, and can not support the secretion of E2 and progesterone [23]. These would partially explain why the LPD happen and why LPS is required. In the absence of LS, premature luteolysis result in decreased level of progesterone [24].

### Luteal phase support (LPS) in the fresh embryo transfer cycle

#### LPS in the cycle with hCG trigger

As hCG and LH activate the same receptor, hCG (5000–10,000 IU) can induce final oocyte maturation, and maintain CL function for about 5 days because of its longer half-life time [25]. At the time of embryo implantation, the level of hCG originating from the ovulation trigger begin to decrease, which will negatively affect the CL producing endogenous progesterone. Therefore, it is essential to supplement progesterone from this time to a point when endogenous hCG was secreted by the implanted embryo [26].

Although the COS caused the disordered production of LH, the final result is a lack of CL support. Progesterone is the natural alternative that compensates the luteal defection caused by COS in ART. The preparations of progesterone are discussed in the above part of this review.

Progesterone is maily producted by the CL. Animal studies with sheep showed that the average progesterone level in the ovarian vein was 800 times higher than the average level in jugular vein [26]. Therefore, endogenous progesterone within CL may not only raise the systemic circulating concentration but also have a local direct effect on the uterus.

Therefore, the gap in LH-like activity can be covered by continuously providing a low dose of hCG (500 IU) so that in the middle luteal phase, the hCG will increse slightly to about 9 IU/L.

However, using hCG as LPS may have disadvantages. By its VEGF-triggering effects on oavry, hCG leads to the fluid shifts, which was the characteristic of OHSS [26].

#### LPS in the cycle with GnRH-a trigger

In patients with antagonist protocol [26–28] or other non-downregulation protocols, GnRH-a has been used for trigger. The affinity coefficient of GnRH-a for the GnRHR is 2–5 times higher than that of endogenous GnRH [29]. This induces endogenous peak of FSH and LH, but the magnitude of LH activity is lower than hCG trigger and natural cycle [30]. Without LPS, the mean life span of CL after GnRH-a trigger was only 4 days, and was 13 days after hCG trigger [19, 31]. Thus, a distinguishing feature of the GnRH-a trigger is that it separates two events: the induction of final oocyte maturation and the suport of the CL in eary LP [32, 33].

The early studies investigating GnRH-a triggering followed by conventional LPS demonstrated an unacceptably low implantation rate [26, 34]. Since embryo qualities are comparable, as well as the implantation rate in oocyte donation cycles and in FET cycle was not hampered, a conclusion was drawn that the problem is an abnormal luteal phase. Previous studies have hypothesized that all women will suffer severe luteolysis within about 5 days after GnRH-a trigger [21]. But, it has recently been clearly showed that luteolysis is patient specific [22, 35]. Until now, two different modifed LPS protocols [36] has been presented.

### The American approach

The American approach means supplementation with both E2 and progesterone and adjust doses as needed according to serum steroid levels.

This LPS protocol has been reproded [37] in a RCT including 66 women with PCOS or high response. Intensive LPS begins with 50 mg i.m. progesterone q.d and E_2_ 0.3 mg transdermaly q.o.d. Serum E2 and progesterone levels were assessed 3–7 days after oocyte pick-up and weekly thereafter, and hormonal supplementation continued until approximately 10 week of pregnancy. Based on serum levels, a maximum of 75 mg progesterone can be used per day, and other progesterone was added to maintain progesterone > 20 ng/mL. Similarly, E2 could be added to 0.4 mg q.o.d, and oral E2 (2 mg to 8 mg) is added to maintain E2 > 200 pg/mL. As a result, the ongoing pregnancy rate was higher with intensive LPS than that with standard LPS (53% versus 48.3%).

Enegmann et al. [38] performed a research of infertile women with high OHSS risk and E2 ≤ 4000 pg/mL. Women received a double trigger (leuprolide acetate 1 mg + 1000 IU hCG) combined with intensive LPS, showing a higher implantation and live birth rates compared with GnRH-a trigger alone. In this study, patients with a maximum serum estradiol level > 4000 pg/mL with only GnRH-a trigger received intensive LPS, and gain satisfactory pregnancy outcome. It is worthy noting that other studies using similar intensive LPS also reported favorable results after fresh transfers [39–41].

Increasing evidence showed that E2 supplementation as LPS does not bring beneficial effect [42, 43]. Thus it was suggested E2 especially with large dose might not be necessary.

### The European approach

#### Dual trigger with hCG

Shapiro et al. [44] reported the combination of GnRH-a and low-dose hCG trigger for the first time. Based on patients’ body weight and risk of OHSS, they used leuprolide acetate 4 mg and hCG ranging from 1000 to 2500 IU. In the end the study showed a higher pregnancy rate, but the incidence of OHSS was also increased with higher dose hCG. The same author later published a further study, reporting an ongoing pregnancy rate of 57.7% in patients underwent dual trigger, with only one case of OHSS [41]. To reduce the risk of OHSS, dual trigger with 1000 IU hCG and GnRH-a following with intensive LPS was proposed. The live birth rate was significantly higher than GnRH-a trigger alone (52.9% versus 30.9%) in the case of E2 < 4000 pg/mL [45]. Another benefit of dual trigger is that it can be used as a ‘‘backup’’ in the event of GnRH-a trigger failure [46].

#### Low-dose hCG at time of oocyte retrieval

A single injection of 1500 IU hCG on the oocyte pick-up day apart from standard LPS has been reported by Humaidan et al. in a number studies [27, 47, 48]. In a study with 302 IVF cycles, they compared the administration of 1500 IU hCG after GnRH-a trigger with hCG trigger, and found similar delivery rates [48]. A retrospective studies found that the clinical pregnancy rate was 41.8%- 52.1%, while the incidence of severe OHSS was maintained at a low level [39, 49]. Two studies [39, 49] reported that, in women at high risk of OHSS, 1 out of 71 and 2 out of 275 severe OHSS cases after receiving 1500 IU hCG on the oocyte pick-up day. However, Seyhan et al. reported that the incidence of severe OHSS reached to 26% [50] in 23 women at high risk of OHSS recerving GnRH-a for trigger and hCG (1500 IU) on oocyte pick-up day.

Many studies have explored the appropriate timing to add low dose hCG. A well-designed clinical trial compared pregnancy rate and incidence of OHSS in high-risk women when low-dose hCG was administrated at GnRH-a trigger day (group 1) or 35 h later (group 2) [51]. There was a similar live birth rate (53.8% versus 61.3%; P = 0.57). Compared with group 1, the incidence of OHSS in group 2 was slightly higher without significant difference (9.7% versus 3.8%; P = 0.62). Therefore, either protocol is reliable for women with high risk of OHSS. Lower dose of hCG administered earlier may result in decreased incidence of OHSS.

Another RCT compared 1500 IU hCG 12 h with 35 h after GnRH-a trigger following by standard LPS [27]. P level was significantly higher in the 35 h group compared with the 12 h group. The clinical pregnancy rate were similar between the 35 h group and the hCG trigger group, and significantly higher than that of the 12 h group. Therefore, the optimal timing for low dose hCG injection seemed to be at 35 h after GnRHa trigger.

#### Low-dose hCG in the luteal phase

Haas et al. reported that 5 women who received 1500 IU hCG 3 days after oocyte pick-up had significantly higher progesterone levels than 6 women without hCG after oocyte retrieval. The pregnancy rates were similar between groups, and no severe OHSS was reported [52].

Castillo et al. [53] explored the effect of low-dose hCG administration intermittently after GnRH-a trigger in 192 women at high risk of OHSS. They were given 1000 IU, 500 IU, or 250 IU hCG every 3 days from oocyte pick-up day. The clinical pregnancy rate was 43.4% and the incidence of moderate and severe OHSS was 4.1% and 3.6%, respectively.

#### Exogenous progesterone -free LPS

After the GnRH-a trigger, the LPS with solely exogenous hCG without exogenous progesterone was first reported by one study. This study included 15 normal responders who failed to pregnance in previous cycle with hCG trigger. After GnRH-a trigger, only two 1500 IU hCG was given on the day of oocyte retrieval and 3 days later. According to reports, the ongoing pregnancy rate was 47%, and no OHSS occurs in these low-risk patients [54]. In order to conduct more studies on hCG based LPS without exogenous progesterone, two pilot RCTs were performed on IVF patients with normal ovarian response triggered with either hCG or GnRH-a [16, 55]. In the GnRH-a trigger group, a small amount of subcutaneous hCG (125 IU) injection was given for 14 days. In contrast, the other group received standard LPS. The ongoing pregnancy rate was 42% and 39% for GnRH-a and hCG trigger in one study respectively, while 38% and 41% in the other study.

The introduction of the exogenous progesterone -free LPS is an innovation. After triggering with GnRH-a, the CLs will be down regulated and a low dose of hCG can partially restored the CLs function. In contrast, hCG has a longer half-life, and can support the CLs functionally within a few days after hCG trigger. This LPS protocol has become a new method. However, it should be caution when using this protocol after hCG trigger due to the increased risk of OHSS [56, 57]. High risk of severe OHSS would be considered as a relative contain indication of this LPS protocol.

#### rLH luteal supplementation

Repeated administration of rLH is another method of increasing LH activity.

Papanikolaou [58] conducted a study in which 300 IU rLH were given on the day of OPU, OPU + 2, OPU + 4, OPU + 6, OPU + 8 and OPU + 10—besides standard LPS in GnRH-ant cycles after GnRH-a trigger. The control group used hCG to trigger. Compared to the standard protocol, the novel rLH luteal supplementation regimen achieved similar implantation rate and delivery rate. No OHSS happened in either group, but study with larger sample size was required to ensure the effect of rLH for LPS. Besides, the cost efficacy should be taken into consideration as well.

#### GnRH-a for LPS

Pirard et al. conducted three studies, which investigated the GnRH-a administration as a replacement for progesterone as LPS. These three studies have shown that GnRH-a administration continuously alone for LPS is effective in the non-downregulated cycle [59–61].

Recently, a retrospectively study with 2529 ART cycles evaluated the efficacy of GnRH-a as sole LPS compared with standard LPS with vaginal progesterone in GnRH-ant cycles. LPS stoped 2 weeks after oocyte pick up if the hCG result is positive. The results indicate that intranasal GnRH-a daily for LPS is associated with a better pregnancy outcome than the traditional LPS with vaginal progesterone [62].

The progesterone and E2 levels were higher in the GnRH-a group. This may be the possible explanation for favorable pregnancy outcome [63]. In addition, the nasal spray of GnRH-a for LPS was a more convenient method compared to the currently used LPS, avoiding irritation of vaginal preparations and pain of injection.

### Unanswered questions

#### Initiation of luteal phase support

Nowadays, no one would question the necessity for LPS in COS. However, there is still doubt as to when it should be initiated. In the hCG triggered IVF cycle, the production of progesterone after trigger continues until 5–6 days after oocyte retrieval [7]. In rLH or GnRH-a triggered cycle, the initial drop of progesterone from the CL was even faster [7]. Compared with the GnRH-a or rLH trigger, the endogenous progesterone level generated by the hCG trigger is higher and lasts longer.

Early administration of progesterone was proposed to be beneficial by the relaxation effect of progesterone on uterine smooth muscle [64]. However, COS alway casue the endometrium dating in advance, resulting in the asynchrony between embryo and endometrium and embryo implantation failure, and supplementation of progesterone too early would aggravate this phenomenon [65].

A well-designed study found that progesterone administration 12 h before oocyte retrieval has a decreased pregnancy rate compared with the start of LPS on the day of oocyte retrieval [66]. Mochtar et al. [67] examined optimal initiation of vaginal progesterone in a prospective randomized study: progesterone was provied either on trigger day, on the day of oocyte pick-up, or on the ET day. The ongoing pregnancy rate was lower when start LPS on trigger day, though the difference has no statistically significance. Therefore, starting progesterone supplementation too early may bring detrimental effect on the pregnancy outcome.

A prospective study compared progesterone supplementation started on the 3rd versus the 6th days after oocyte retrieval, and found that initiation of LPS the 3rd days after retrieval, leading to a higher pregnancy rates [68]. The reason is that the stimulation to CL with hCG ends at about day 5–6 after oocyte retrieval. Three studies [69–71] started LPS on the day after retrieval, whereas another two trials [72, 73] started LPS with vaginal progesterone 2 days after oocyte pick-up. One study indicated that progesterone supplementation started 1 day after oocyte pick-up did not reduce the pregnancy rate, implantation rate, or live birth rate in women with the GnRH-a long protocol. In a study of 1111 IVF/ICSI cycles, LPS was given immediately or 4 days after oocyte retrieval, and there was no difference in pregnancy outcomes (Feichtinger et al. 2011). In addition, two studies showed no difference in pregnancy outcomes when LPS was started on the day of oocyte retrieval or on ET day (day 2 or day 3) (Feichtinger et al. 2011), [68]. Investigators conducted a small sample RCT, and found that no matter the progesterone administration started on the evening of oocyte retrieval or on the evening of ET, the embryo implantation and pregnancy rates were similar [74, 75]. A recent meta-analysis [68] summarized the timing of the LPS in ART, and confirmed that starting LPS before oocyte retrieval was associated with a significant reduction in pregnancy rate; In contrast, there was no difference in the pregnancy rate when LPS was started on the day of oocyte retrieval or 1 ~ 3 days later. Three systematic review [68, 76, 77] indicated that most IVF centers begin to provide progesterone supplementation betwen oocyte pick-up and ET.

#### Duration of luteal phase support

In addition to the initial of LPS, the duration of LPS has not yet been widely agreed to reach a consensus [78]. In the case of ovulation, the luteal-placental shift does not happen until 8–10 week of pregnancy. Studies have shown that the level of progesterone is 75% at the 6th week of pregnancy and reduced to 50% and 25% at the 10th and 15th week of pregnancy, respectively. The production of progesterone by the placenta increased significantly after 8 weeks of pregnancy, so the luteal-placental shift began at this point [79].

Therefore, the LPS were provided until about the 10th week of pregnancy in the clinical practice. After that time, data about ovariectomy indicated that the function of CL is not necessary for the maintenance of pregnancy [79]. Even women with ovaries absent or blocked can become pregnant successfully, this experienc confirmed that there is no need to seek LPS anymore [80]. Conversely, there is growing evidence that LPS can be discontinued by the 10th week of pregnancy in ART [81]. Recently, one study summarized the currently used LPS protocols via conducting a questionnaire survey in 1480 clinicians all over the word [82].

A prospective study [83] showed that vaginal progesterone supplement as LPS can be safely discontinued at 5w of pregnancy, having a similar outcome to LPS up to 8w of pregnancy. Aboulghar and colleagues conducted a research in which patients were indided to either continue or discontinue receiving progesterone when there was fetal heart with ultrasound, and found comparable outcomes between the groups, indicating that continuing progesterone beyond this time has no benefit [78]. In addition, several studies have found no difference in pregnancy outcome and suggested that progesterone supplement would be safely discontinued after the first test for positive β-hCG [84, 85].

A meta-analysis assessed the optimal duration of progesterone supplementation after IVF/ICSI, and concluded that it was unnecessary to continue progesterone supplementation after the first hCG test [86]. They called for RCT with large sample size to clarify the duration of LPS after ART.

Another large-scale survey of 84 reproductive centres from 35 countries was conducted recently, encompassing 51,155 cycles. The result showed that 67% cycles discontinued progesterone at 10–12 weeks of gestation, 22% cycles discontinued LPS when fetal heart appeared and 12% discontinued LPS when the test of hCG test was positive [87]. It is generally believed that continuous LPS is better than taking a risk of miscarriage with earlier discontinuation [87–89]. However, infertile couples usually under great pressure both physiologically and psychologically, individual LPS can decrease adverse effects of over-treatment and reduce the psychological and financial burden.

## Conclusion

**Fig. 1:**
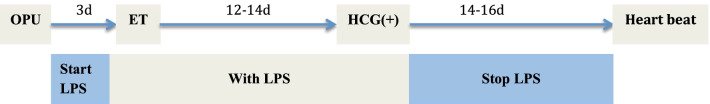
Optimal initiation and duration of LPS. The optimal initiation of LPS should be between 24 and 72 h after oocyte retrieval; the duration of LPS should at least last to pregnancy test is positive. *OPU* oocyte pick-up, *ET* embryo transfer, *LPS* luteal phase support

It is our responsibility to provide individualized LPS for infertile women base on their specific characteristics, desires and the treatment protocol. It is recommended to initiate the LPS between 24 and 72 h after oocyte retrieval and continue at least until the hCG test is positive (see Fig. 1). The addition of E2 and the route of progesterone administration appear to be independent of the improvement in outcomes.

## Data Availability

The datasets supporting the conclusions of this article are included within the article.

## Abbreviations

IVF: In vitro fertilization
ICSI: Intracytoplasmic sperm injection
ART: Assisted reproductive technology
LPS: Luteal phase support
COS: Controlled ovarian stimulation
LPD: Luteal phase deficiency
OHSS: Ovarian hyperstimulation syndrome
GnRH-a/ant: Gonadotropin- releasing hormone agonists/antagonist
CL: Corpus luteum
E2: Estradiol
P: Progesterone
OS: Ovarian stimulation
GCs: Granulosa cells
IR: Implantation rate
PR: Pregnancy rate
